# Poster Session II - A240 DIETARY ARABINOXYLAN FIBER INTAKE ASSOCIATED WITH ANTIMICROBIAL HUMORAL RESPONSES AND DECREASED CROHN’S DISEASE RISK

**DOI:** 10.1093/jcag/gwaf042.240

**Published:** 2026-02-13

**Authors:** S Jeong, B Thakur, C O Elson, R Khorasaniha, A Waslyk, Q Zhao, S Lee, A Griffiths, H Steinhart, L A Dieleman, H Huynh, G Aumais, R Panaccione, H Armstrong, K Croitoru, W Turpin

**Affiliations:** Lunenfeld-Tanenbaum Research Institute, Sinai Health, Toronto, ON, Canada; Lunenfeld-Tanenbaum Research Institute, Sinai Health, Toronto, ON, Canada; University of Alabama at Birmingham Health System, Birmingham, AL; Lunenfeld-Tanenbaum Research Institute, Sinai Health, Toronto, ON, Canada; Lunenfeld-Tanenbaum Research Institute, Sinai Health, Toronto, ON, Canada; University of Alabama at Birmingham Health System, Birmingham, AL; Lunenfeld-Tanenbaum Research Institute, Sinai Health, Toronto, ON, Canada; SickKids Research Institute, Toronto, ON, Canada; Lunenfeld-Tanenbaum Research Institute, Sinai Health, Toronto, ON, Canada; University of Alberta, Edmonton, AB, Canada; University of Alberta, Edmonton, AB, Canada; Hopital Maisonneuve-Rosemont Centre de Recherche, Montreal, QC, Canada; University of Calgary, Calgary, AB, Canada; University of Alberta, Edmonton, AB, Canada; Lunenfeld-Tanenbaum Research Institute, Sinai Health, Toronto, ON, Canada; Lunenfeld-Tanenbaum Research Institute, Sinai Health, Toronto, ON, Canada

## Abstract

**Background:**

Crohn’s disease (CD) is a chronic inflammatory bowel disease thought to arise in part from an augmented immune response to gut microbes. Elevated antimicrobial antibodies towards gut microbes can be detected up to 10 years before symptom onset, independent of other risk factors. Dietary fiber is associated with reduced CD risk, but the contribution of specific fiber subtypes to early immune dysregulation is poorly understood.

**Aims:**

To determine whether individual dietary fiber subtypes are differentially associated with pre-CD antimicrobial responses, B-cell abundance, and CD risk.

**Methods:**

We analyzed 333 CCC-GEM participants (66 pre-CD, 267 controls) as a nested case-control cohort matched by age, sex, follow-up duration and location. At recruitment, participants filled in a food frequency questionnaire and provided serum samples. Using fiber databases incorporating published values we estimated the caloric-adjusted intake of five fiber subtypes. Serum samples were individually probed against 49 gut bacteria flagellins and analyzed for IgG responses. Circulating mucosa homing B-cell clusters (CD19+, CD20+, CCR6+/α4β7+) were identified using mass cytometry. Associations were tested using conditional regression. Using the entire cohort, time to CD onset was assessed using a Cox model.

**Results:**

Among the five dietary fiber subtypes, only arabinoxylan (AX), was associated with reduced antimicrobial immune responses. Of 49 IgGs, 15 were negatively correlated with AX intake (β range -0.21 to -0.11, p < 0.05), 10 of which targeted *Lachnospiraceae* flagellins. Furthermore, we identified five mucosa homing B-cell clusters across participants. Similar to the IgGs, the abundance of three out of five B-cell clusters was negatively associated with AX intake. Given the unique findings for AX, we investigated AX intake’s influence on CD risk across the whole cohort and found that low AX intake was associated with increased CD risk (HR = 3.10, p = 0.008).

**Conclusions:**

Unlike other dietary fiber subtypes, AX intake was consistently associated with lower antimicrobial IgG responses, reduced B-cell counts, and decreased CD risk. These findings confirm that not all fibers confer equal immunomodulatory benefits. AX, found primarily in cereal grains, may represent a diet-based preventive strategy for high-risk individuals to prevent the development of CD.

**Funding Agencies:**

CCC, CIHRHelmsley Charitable Trust

